# An integrated understanding of the impact of hospital at home: a mixed-methods study to articulate and test a programme theory

**DOI:** 10.1186/s12913-024-10619-7

**Published:** 2024-02-02

**Authors:** Hong Chen, Agnieszka Ignatowicz, Magdalena Skrybant, Daniel Lasserson

**Affiliations:** 1 Warwick Medical School, Gibbet Hill Campus, University of Warwick, Coventry, CV4 7AL UK; 2 Murray Learning Centre, Institute of Applied Health Research, University of Birmingham, Birmingham, B15 2TTT UK; 3 Department of Geriatric Medicine, Oxford University Hospitals NHS Foundation Trust, John Radcliffe Hospital, Oxford, OX3 9DU UK

**Keywords:** Hospital at home, Acute care, Older people, Multidisciplinary care, Person-centred care, Patient benefit, Programme theory, Organisation, Programme impact theory, Mixed methods

## Abstract

**Background:**

Hospital at Home (HaH) provides intensive, hospital-level care in patients’ homes for acute conditions that would normally require hospitalisation, using multidisciplinary teams. As a programme of complex medical-social interventions, a HaH programme theory has not been fully articulated although implicit in the structures, functions, and activities of the existing HaH services. We aimed to unearth the tacit theory from international evidence and test the soundness of it by studying UK HaH services.

**Methods:**

We conducted a literature review (29 articles) adopting a ‘realist review’ approach (theory articulation) and examined 11 UK-based services by interviewing up to 3 staff members from each service (theory testing). The review and interview data were analysed using Framework Analysis and Purposive Text Analysis.

**Results:**

The programme theory has three components- the organisational, utilisation and impact theories. The impact theory consists of key assumptions about the change processes brought about by HaH’s activities and functions, as detailed in the organisational and utilisation theories. HaH teams should encompass multiple disciplines to deliver comprehensive assessments and have skill sets for physically delivering hospital-level processes of care in the home. They should aim to treat a broad range of conditions in patients who are clinically complex and felt to be vulnerable to hospital acquired harms. Services should cover 7 days a week, have plans for 24/7 response and deliver relational continuity of care through consistent staffing. As a result, patients’ and carers’ knowledge, skills, and confidence in disease management and self-care should be strengthened with a sense of safety during HaH treatment, and carers better supported to fulfil their role with minimal added care burden.

**Conclusions:**

There are organisational factors for HaH services and healthcare processes that contribute to better experience of care and outcomes for patients. HaH services should deliver care using hospital level processes through teams that have a focus on holistic and individually tailored care with continuity of therapeutic relationships between professionals and patients and carers resulting in less complexity and fragmentation of care. This analysis informs how HaH services can organise resources and design processes of care to optimise patient satisfaction and outcomes.

**Supplementary Information:**

The online version contains supplementary material available at 10.1186/s12913-024-10619-7.

## Background

Hospital at Home (HaH) is a service that provides acute and subacute care by healthcare professionals in private or care homes for a condition that would otherwise require acute hospital inpatient care [1]. The severity of the condition managed differentiates HaH from other community service provision as well as the specialist nature of the senior decision-makers [1, 2]. It covers short, time-limited acute episodes of care: patients are treated as though admitted to hospital but managed within their own home. It is delivered by multidisciplinary teams of healthcare professionals complying with current acute standards of care. It treats people with a wide range of conditions in a variety of contexts. Nevertheless, a common feature is the acuity and/or the complexity of the patient’s condition—often associated with older age and frailty [1, 3–9].

Generally speaking, there are two types of HaH: Admission Avoidance HaH provides acute and/or subacute care in a patient’s (care) home to avoid admitting the patient to hospital as an inpatient [10], and Early Discharge HaH supports patients who have already been admitted as an inpatient to go home earlier than usual to complete acute and/or subacute care in their home, thereby reducing the length of hospital stay [11]. However, the range of patient populations encompassed, the specification of interventions, including the way in which services are accessed and the scope and intensity of healthcare professional input, vary widely worldwide [1, 9]. The evidence base for HaH interventions is thus characterised by heterogeneity. A recent systematic review of reviews [9] and two Cochrane systematic reviews of randomised trials [10, 11] suggest that, for suitable patients, HaH services (both types) may provide either superior or similar outcomes compared to inpatient care, based on mixed evidence with low to moderate certainty. More specifically, HaH probably makes little or no difference in risk of death or likelihood of hospital readmission compared to inpatient care [3, 9–12]. HaH patients may have lower risks of hospital-acquired infections and functional decline- both physical and cognitive [8, 13, 14]. For older patients, it may reduce the likelihood of transfer to a care home following an acute episode [1, 7, 10, 11]. Cost-saving may be derived from shorter length of stay, lower use of clinical testing and consultations, and reduced admissions and readmissions [3, 6, 15]. Nonetheless, HaH patients generally experience high levels of satisfaction with the service [4, 9, 10, 12, 16–18] and appreciate having: comfort in their home environment; ease of admission processes and convenience of care; feelings of safety, reassurance and appreciation; a more seamless care experience with fewer gaps in care transition; greater control over treatment; increased sense of independence; (perceived) quicker recovery; and better physical activity, sleep quality, mood and social contact [3, 4, 16, 19].

Aiming for person-centred care, HaH provides multidisciplinary, coordinated care in the home, working with patients and carers and interfacing with existing acute and also community-based health and social care services [1]. It is therefore inherently complex, with multiple, interacting strands of activities/interventions delivered by different professionals at multiple levels through complex relationships and interactions within and across professional and organisational boundaries [20]. Flexibility and adaptability to individual needs/circumstances and local contexts are its strength which also entails variations in the service model [21, 22]. The UK national policy on virtual wards supports the rollout of HaH [23]. However, there is a lack of clarity on the essential activities, functions, and processes intended for the organisation and delivery of HaH and how these impact on patients, cares and beyond.

A programme theory in healthcare, the foundation on which every programme rests, is a conceptual model of how a programme is expected to work and the connections presumed between its various activities and functions and the patient and other benefits it is intended to produce [24]. A sound theory can facilitate the design, long-term feasibility and implementation of healthcare services and positively impact on evaluations of the services. For example, in a realist review to identify, develop and refine programme theory for intermediate care, the broad mechanisms that occurred at service user, professional and organisational levels were identified by the review team [25]. The resulted programme theory provided a ‘road map’ of the complex set of factors that decision-makers should consider, to make intermediate care as effective as possible in any given local context. According to the authors, the theory could also be used as a ‘diagnostic checklist’ to highlight weaker areas of existing intermediate care provision for improvement, or as a stimulus for measuring the extent to which a service addresses these factors within a local care context. In addition, the progress made by the review towards the specification of mechanisms at individual and organisational levels could also inform the focus of future research.

HaH being a programme of complex medical-social interventions, a sound theory is needed to support its service development, monitoring and evaluation, and strategic and policy planning. Programme theory is implicit in a programme’s structure and activities [24]. In recent years, various types of evidence have started to emerge, shedding light on the organisational, operational and implementation issues, or how the personal, social, clinical and technological aspects of HaH and interactions among these led to the patient outcomes. Our study aimed to extract the tacit theory from this body of evidence and draw on interviews with UK HaH professionals to test the soundness of the theory. This article focuses on programme impact theory, which links effective care delivery and utilisation to the intended benefits, showing multiple, interacting pathways of change.

## Methods

This section reports on the first two components of a five-component mixed-methods study, i.e. literature review and professional interviews (see Supplementary File 1). The aims of these two components were to articulate and test a HaH programme theory respectively.

### Model of programme theory

**Fig. 1:**
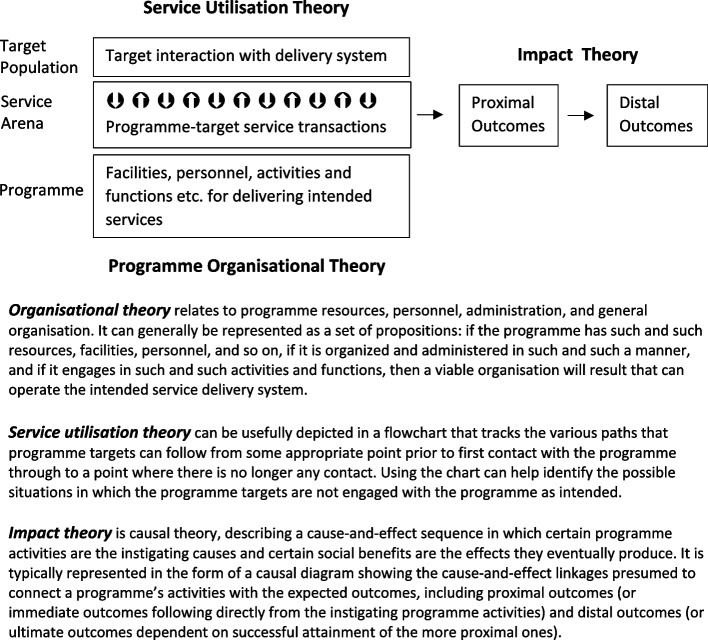
Overview of our model of programme theory. Source: Adapted from Rossi and colleagues’ definition of programme theory [24]

Programme theory has been described and used under various names, for example, logic model, program model, outcome line, cause map, action theory, change theory [24]. There is no general consensus about how best to describe a programme’s theory. We found Rossi and colleagues’ scheme (summarised in Fig. 1) the most useful for this research, which “depicts a social programme as centring on the transactions that take place between a programme’s operations and the population it serves”, highlighting three interrelated components of a programme theory: impact theory, utilisation theory, and organisational theory [24]. It was a useful guide for the data collection and analyses (as described below) and the model on which we built our programme theory (as reported in the Results section).

### Literature review (theory articulation)

The review aimed to unearth programme theories that implicitly or explicitly underpin HaH’s families of interventions. A realist review approach was taken because of its explanatory rather than judgemental focus and because it adopts a qualitative systematic review method whose goal is to identify and explain what it is about this programme that works, for whom and in what circumstances [26]. However, driven by the aim and the design of the research as well as the time and funding constraints (one-year, rapid-response research with multiple, sequentially and conceptually linked components), this review has only gone so far as to pursue the initial phase of a classic realist review: “theory stalking and sifting” [26].

**Fig. 2:**
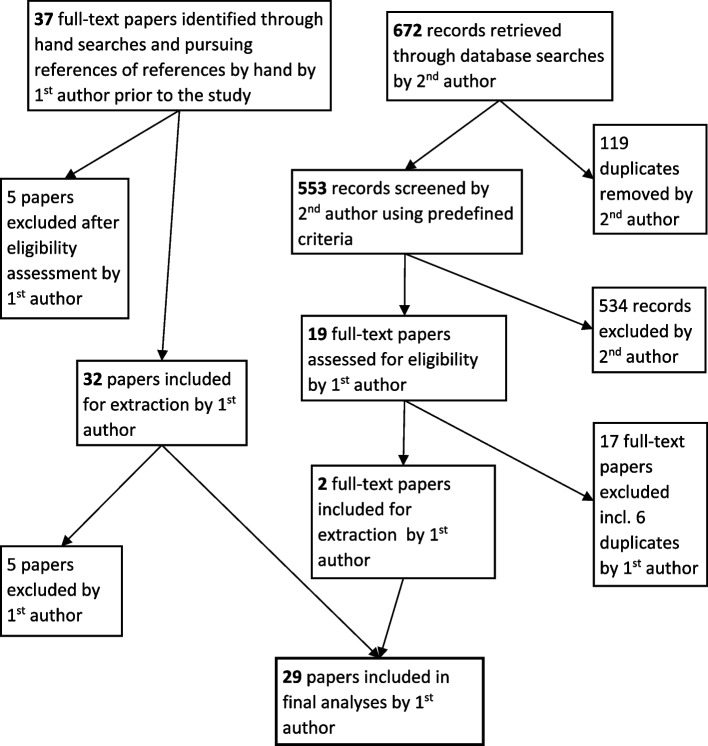
Flow chart of source identification for inclusion

The search of evidence was purposive and theoretically driven with the explicit purpose of collecting data that relate not to the efficacy of interventions, but to the range of prevailing theories and explanations of how interventions are supposed to work and why things go well or wrong [26]. As such, different types of information and evidence were searched and included, with value placed on qualitative studies and grey literature so as to identify vital explanatory ingredients. Multiple search strategies were used including snowballing, hand searching, and database searching. More specifically, prior to the study, the first author (HC) had accumulated a collection of suitable literature (37 papers) through hand searches and snowballing during the process of the grant application. During the study, the second author (AI) carried out searches in three databases using predefined key words and certain limits (see Table 1 below and Supplementary File 2 for more details), which resulted in 6 duplicates (already included in the above-mentioned 37 papers) and two extra papers being identified (Fig. 2).

**Table 1:** Database searches

**Databases:** Medline, Embase, HMIC
**Search terms:** hospital at home; rapid response (team); acute care at home; hospital in the home; home hospitalization; hospital‐based home care
**Search limit:** No restrictions are applied regarding types (e.g. reviews, commentaries, editorials, grey literature, evaluations). Method is restricted to qualitative and mixed methods with a qualitative component. Language is restricted to English. Date is limited to 2015 to May 2021

Pawson and colleagues’ realist principle on quality assessment was adopted [26], i.e. the worth of a source was to be established in synthesis- not on the grounds of rigour. According to the authors, in realist reviews, all sources can be both flawed and illuminating. Different sources can contribute different elements to the rich picture that enables the theory articulation. The limitations of one source often can be met with information from another. The results of one can be explained by the findings from another. As such, we did not use critical appraisal checklists to assess the rigour of different types of evidence included. Sources were only considered in respect of whether and how much they could contribute to main types of information needed to articulate theory, i.e. fitness for explanatory purpose, as guided by our chosen model of programme theory (Fig. 1) and as specified in the next paragraph.

We used Nvivo 12 to extract data from 34 sources: relevant information was directly coded (gathered) into nodes (very broad factual and conceptual categories at this stage) as if it was qualitative data from primary research. Four main types of information were extracted (coded) from the sources, as they were deemed particularly useful in “theory stalking” [24]: programme goals and objectives; programme components, functions, and activities; outcomes including process outcomes, patient and carer outcomes and service and system related outcomes; temporal sequencing and logical or conceptual linkages among functions, activities, components and outcomes. More broadly, we also paid attention to the building blocks of health systems: governance, information, financing, service delivery, human resources, and medicines and technologies [27]. After weighing up the relative contribution of each source, 5 sources were dismissed because of their limited contribution. This resulted in 29 articles included into the final synthesis (see Supplementary File 3).

We used Framework Analysis [28] to identify commonalities and differences in the data as well as relationships between different parts of the data, thereby seeking to discover descriptive and explanatory findings clustered around themes. The initial codes and the final analytical framework (themes and subthemes) developed by the first author were validated by members of the multidisciplinary research team through Intercoder agreement [29] and audit trail [28], and by public contributors through a public involvement workshop. Additionally, we conducted Purposive Text Analysis [30] to micro-analyse the arguments made in the literature (i.e. causal and dynamical depictions) about HaH services’ structure and behaviour and the subsequent results and effects. This analysis resulted in a causal diagram showing the cause-and-effect linkages presumed to connect a programme’s activities with the expected outcomes and impact, i.e. the impact theory (see Fig. 1). More specifically, this method employed an entirely inductive approach to identify problems, key variables, and their structural relationships from qualitative data. The core analytical steps included: a) identifying data segments that consisted of one argument and its supporting rationales; b) from each data segment, identifying the cause variable, effect variable, and the polarity of the relationship; c) using simple words-and-arrow diagram to represent each causal relationship; d) collecting and merging the words-and-arrow diagrams into a collective causal diagram, collapsing similar variables using a common variable name. We used a specialised, system dynamics software- Vensim PLE (https://test.vensim.com/causal-tracing/) (often used to produce Causal Loop Diagrams) to aid this complex process and construct the impact theory (reported in the Results section).

The ideas unearthed in the above analyses were many and varied (see Supplementary File 4). They stretched from macro theories (e.g. health inequalities) to meso theories (e.g. organisational capacity) to micro theories (e.g. employee motivation). The final task was to decide upon which combinations and which subset of theories were going to feature in the final integrated theory and how these could be represented. We aimed to find a level of abstraction that would allow the researchers to stand back from the detail and variation in the evidence, but that would be also specific enough to meet the purpose of the review- to inform practice and policymaking. The theory development process was iterative and complex, involving, for example, deconstructing interventions into component theories, changes from framework building to framework testing and from theory construction to theory refinement using the same data, and a shift from divergent to convergent thinking as ideas began to take shape and the theories underpinning the intervention gained clarity [24].

### Professional interviews (theory testing)

We aimed to test the theory and capture lessons learnt on implementing HaH services (the latter to be reported elsewhere). A programme theory involves many assumptions about how things are supposed to work that can be assessed by observing the programme in operation, talking to staff and service recipients, and making other such inquiries focused specifically on the programme theory [24]. In this study, we chose to interview HaH staff from different services in the UK to assess how plausible and realistic the programme theory is, as part of a rapid-response research. A topic guide was developed based on general literature on the evaluation of health service implementation as well as the review findings (see Supplementary File 5). It was designed to collect data to test the theory and capture lessons learnt (the latter to be reported elsewhere).

Purposive sampling was employed [31]: we recruited the National Health Service (NHS) staff who had had experience in designing, planning and/or delivering HaH service through a professional Society- The UK Hospital At Home Society. The Society aimed to raise awareness of the patient and healthcare provider benefits that HaH can offer as well as benchmarking best practices and providing practical advice for setting up HaH care. Most members of the Society were NHS practitioners who were involved in developing and delivering HaH and some were considering doing so. Altogether 190 registered members were invited twice, 39 expressed interest, and 16 signed up for the study. As we solely used online methods to interact with participants, informed consent was obtained by return emails, which were then retained including the header information with emails addresses and dates (see Supplementary File 6).

Between 13th and 22nd September 2021, we conducted either small group (2–3 participants) or individual professional interviews as per participants’ choosing. Altogether 11 interviews (average 50 min per interview) were conducted with 16 professionals (including doctors, nurses, service leads and therapists) from 11 service models (see Supplementary File 7). In other words, we studied 11 HaH services by interviewing up to 3 staff members from each HaH team. These services had been in operation for varying lengths of time—between several weeks and over 10 years by the date of the interview.

The interviews were recorded via Microsoft Teams and the recordings transcribed verbatim. Transcripts were anonymised- participants assigned a unique ID code and other distinguishing features removed. Data were analysed thematically using Framework Analysis [28], with the aid of NVivo12. The first author (HC) coded and analysed the data and findings were validated by the research team (including public contributors) through presentations and discussions in project meetings. The thematic framework (themes and sub-themes) developed for the literature review was the basis for the coding and the analysis at this stage but extended to include themes emerging from the interview data. In this way, we were able to compare the findings from the interviews with those from the literature review centring around the theory, thereby testing the soundness of the theory.

## Results

We have articulated a HaH programme theory using the literature review and tested the theory using the interviews with UK HaH healthcare professionals.

**Fig. 3:**
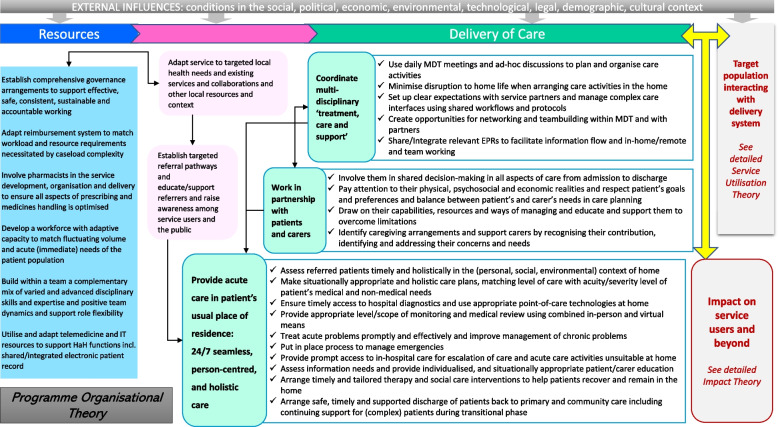
The organisational theory

**Fig. 4:**
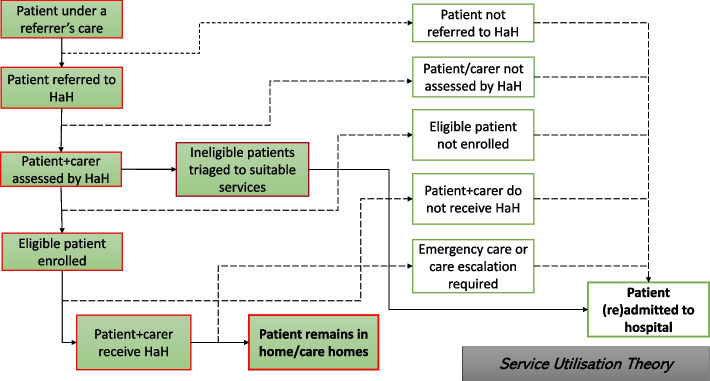
The utilisation theory

**Fig. 5:**
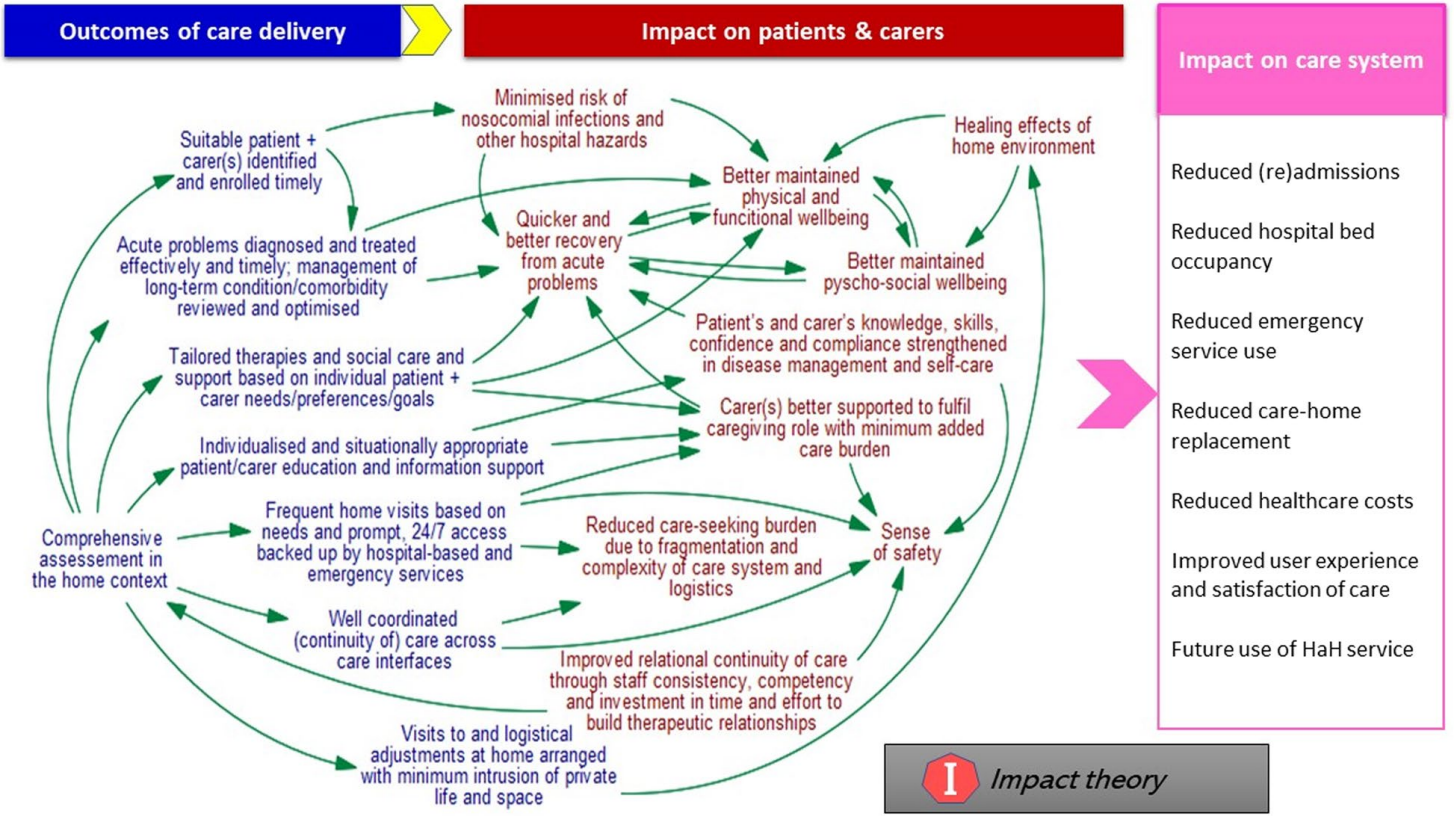
The impact theory

The literature review included 29 articles- mainly research articles, review papers, evaluation reports and service guidelines/manuals, which were published between Jan 2015 and May 2021 by researchers and HaH practitioners from UK, US, Australia, Italy, France, Belgium, Spain and Finland (see Supplementary File 3 for more details). The analyses and synthesis of this body of international evidence resulted in an overarching HaH programme theory consisted of three interrelated components: the organisational theory, the utilisation theory, and the impact theory (Figs. 3, 4 and 5). Together, the three component theories provided an overview of the essential “ingredients” and processes intended for the organisation and utilisation of a HaH service and the impact of this new model of acute care on patients and carers and beyond- as compared to traditional hospital admissions.

Altogether, we studied eleven UK HaH services (two in Scotland, two in Northern Ireland, and seven in England), by interviewing up to 3 staff members from each HaH team. These services had been in operation for varying lengths of time—between several weeks and over 10 years by the date of the interview. A total of 16 staff members were interviewed (see Supplementary File 7 for basic information about the interviewees). The interview findings about the UK services showed no significant deviations from the theory developed from the international evidence, i.e. the theory still holds, is still sound, capable of explaining what HaH is/does, how it works and why in the UK context.

In this section, we present the three component theories as illustrated in Figs. 3, 4 and 5, with a particular focus on the impact theory (Fig. 5). The organisational and utilisation theories (Figs. 3 and 4) are succinctly described to contextualise the impact theory (Fig. 5), i.e. they explain what has to be done or take place (i.e. HaH’s main functions, activities, processes and interactions with target population) for the intended impact to arise. To support and further explain Figs. 3, 4 and 5, we report the main review findings based on which a draft theory was extracted as well as the interview findings based on which the draft theory was tested. Where the interview findings are similar to the review findings, they are combined to avoid repetition, otherwise they are reported separately.

### The organisational and utilisation theories

According to both the review evidence and the interview data, based on whether or not a patient had already been admitted to hospital as an inpatient, there were two distinct types (pathways) of HaH: Admission Avoidance provided acute and/or subacute care in a patient’s place of residence (e.g. home, care home) to avoid admitting the patient to hospital as an inpatient (full substitution of hospitalisation); Early Discharge supported patients who had already been admitted as an inpatient to go home earlier than usual to complete acute care and/or sub-acute care in their place of residence, thereby reducing the length of hospital stay (partial substitution of hospitalisation) [1, 4, 17, 32–34]. Both in the UK (based on the interview data) and other countries (based on the review evidence), eligible patients were typically referred from multiple sources including: emergency department, acute assessment/observation units (e.g. medical assessment unit, frailty assessment unit, observation unit), hospital outpatient clinics, ambulance services, primary care and community physicians and specialists for Admission Avoidance HaH; and acute hospital wards for Early Discharge HaH [1, 5, 17, 20, 21, 33–43]. Most services provided both types, but services differed in terms of which type and which referral sources were more dominant.

HaH services identified in the review [17, 21, 33–35, 39, 42–45] and those included into the interview study had defined eligibility criteria based on some or all of the following conditions:

- Patient with an acute medical condition that requires inpatient or hospital-level care
- Age threshold (e.g. > 65).
- Primary diagnosis (e.g. COPD, cancer).
- Intensity of care (e.g. maximum daily visits, not requiring the permanent presence of a professional).
- Patient being in a stable state.
- Patient having adequate carer support if not independent.
- Home environment appropriate and free of dangers to patients and professionals.
- Patient and/or his/her family having given their informed consent for the service.
- Patient residing within the geographic catchment area of the service.
- Patient meeting the insurance/funding requirement.

Most services (included in the review and the interview study) intended to treat a broad range of acute conditions except a few that was disease specific, e.g. acute exacerbation of COPD [36], cancer [46]. Services differed in exact pathologies they managed at home; however, a common feature was caseload complexity and vulnerability- associated with older age and frailty [1, 4, 5, 16, 17, 32, 34–36, 38–40, 47, 48]. Indeed, the healthcare professionals who were interviewed frequently used the terms “frail”, “vulnerable” and “complex” to describe their target or actual patient population. For most UK services, older age (over 65–75) was an explicit eligibility criterion, but not on its own and not without exceptions to it. Some UK services also required that patients’ frailty scores be higher than 5 or 6; adults who were younger but had a high frailty score or multiple comorbidities could also be included. A couple of UK services used more relaxed age limits (e.g. over 16 or 18), but the actual patients seen turned out to be mostly older people.

Most international services (based on the review) and most UK services (based on the interview) operated 7 days per week but often not 24 h per day [1, 17, 33, 35–40, 42, 45]. Across these services, there were variations in: a) when and how many hours in a day each service operated; b) when each profession in the multidisciplinary team (e.g. medical staff, nursing staff, therapists) worked within a service; or c) when each element of care (e.g. phone access, admission, home visit) was available. Nonetheless, these services made out-of-hours arrangements by linking HaH with existing services in the community and/or hospital, to ensure that patients had access to appropriate services 24/7. Most services (included in the review and the interview study) provided home visits (for assessment, diagnosis, monitoring and treatment and care), which were made by different professions (e.g. medical, nursing, therapy, paramedic, social work, home aid) available in the team and from external partner services; and daily visits ranged from at least once up to 4 times, adaptable to patients’ needs [1, 4, 5, 16, 17, 21, 33–36, 38–40, 43]. On average, the length of each episode of care ranged between three and seven days, which was similar to the length of hospital stay if the patient was admitted; however, it could be extended further dependent on patients’ needs (e.g. 2 weeks) [1, 5, 17, 33, 36, 39, 43].

According to both the review evidence and the interview data, a multidisciplinary team (MDT) appeared to be essential in delivering high quality, person-centred care to a patient population with complex needs; by integrating different clinical disciplines in one team, HaH was able to offer a holistic approach to addressing the clinical and psychosocial needs of patients and their families [1, 5, 21, 33–35, 37, 39–42, 44–46, 49]. Across services and countries (included in the review and the interview study), the composition of MDT varied and the whole team normally functioned under the guidance of a medical director. Most UK services included in the interview study had an MDT team consisting essentially of medical and nursing staff and allied health professionals, despite variations in team composition and size. All these UK teams had medical cover- provided by hospital consultants/doctors in most cases, or general practitioners in two exceptional cases. Among other staff members involved, pharmacists, advanced clinical practitioners with (mostly) nursing or therapy background, nurses at different bands, physiotherapists, occupational therapists were most common. Advanced clinical practitioners often had prescribing certificates and could prescribe in patients’ homes, additional to doctors and pharmacists. To add to the skill mix of the team, some UK services also included paramedics, social workers, healthcare assistants, physician associates, specialty general practitioners, other therapists (e.g. speech and language, dietetic) and admin staff.

The main functions and activities that HaH was expected to perform and the human, financial, and physical resources required for that performance are presented in the organisational theory (Fig. 3). Specifically, the following main themes were identified and included into the theory: governance [1, 35–37, 44, 45, 50]; financing [16, 34, 37–39, 44–46, 50]; pharmaceutical support [1, 32, 36, 41, 45, 46]; workforce development [1, 5, 20, 21, 33–35, 37, 39–42, 44–46, 49]; technological support [1, 21, 36, 39–41, 43–45, 49, 51]; adaptation to targeted local health needs [17, 21, 33–35, 39, 42–45]; adaptation to local service networks, collaborations and other resources [38, 40, 45, 50]; establishing and maintaining targeted referral networks [1, 34, 37, 48, 51]; coordination of multidisciplinary care [1, 20, 21, 34, 37–41, 43–46, 50, 51]; partnership working with patients and carers [1, 4, 20, 21, 34, 35, 39, 42, 48, 50, 52]; and delivery of person-centred and realistic care [1, 4, 5, 16, 17, 21, 33–36, 38–40, 43]. Figure 3 also demonstrates how the HaH programme’s operation/delivery, utilisation and impact are interlinked. The utilisation theory (Fig. 4) demonstrates how HaH programmes were presumed and expected to reach and recruit the target population, provide and sequence service contacts, and conclude the relationship when services are no longer needed or appropriate.

### The impact theory

The impact theory (Fig. 5) consists of key assumptions about the change process actuated by HaH’s activities and functions and the improved conditions of the targeted population that were expected to result. As illustrated, there are multiple, intertwined cause-and-effect sequences in which certain outputs of the main HaH functions and activities presented above were the instigating causes and certain clinical, health and wellbeing, and system-level benefits were the effects they eventually produced.

In Fig. 5, the summative statements (in blue colour) presented under “Outputs of care delivery” are final *output variables* developed using Purposive Text Analysis [30] and the specialised software (https://test.vensim.com/causal-tracing/), which basically reflect the organisational theory (Fig. 3). The summative statements (in red colour) presented under “Impact on patients & carers” are final *impact variables* developed using Purposive Text Analysis [30] and the specialised software (https://test.vensim.com/causal-tracing/). They are the focus of this section and are directly used as subheadings below to organise the relevant findings that support (evidence) and further explain these statements and their interconnections. These findings represent patients’ and carers’ perspectives based on the research studies, service evaluations and literature reviews that explored service users’ own experiences and perceptions of HaH, and the UK healthcare professionals’ perspectives based on the interviews. Where the interview findings are similar to the review findings, they were combined to avoid repetition, otherwise they were reported separately.

The statements (in black colour) presented under “Impact on care system” suggest *potential or intended* impact of HaH at the system level, based on the summative statements presented under “Impact on patients & carers”. They are not the product of Purposive Text Analysis [30]. They are derived from the findings of this study but also from the findings of a more comprehensive literature review that underpinned our original funding application as well as the research team’s wider knowledge about health and care systems.

#### Minimised risk of nosocomial infections and hazards of hospitalisation

Some carers reported that with HaH, they did not have to worry about patients getting hospital-acquired “bugs and germs” or deal with worsening delirium that could have happened had the patient been in hospital [4, 5, 21, 34, 37, 52]. Some patients reported that they did not have to deal with immobility and a lack of activity (causing physical deconditioning), poor diet and sleep, and other potential hazards of hospitalisation. These findings were confirmed by most UK healthcare professionals who were interviewed.

*“Primary outcome for me is a better quality of care, […] leading to less complications from hospital admission. So less delirium, less sarcopenia, less frailty, less reliance on need for rehab and all those other bits and pieces that […] come as a result of patients coming into hospital.” (M7S1)*

#### Healing effects of home environment

Patients and carers consistently highlighted the comfort that one felt in the familiar setting of one’s own home [4, 16, 34, 37, 40]. HaH patients repeatedly mentioned the benefits of being in the familiar home environment: having all the things one needed and one’s own space to “roam” in, knowing where everything was, and being able to do what one routinely did or one felt like doing; and the generally calmer, more relaxing and private environment of home [4, 52]. Also, patients tended to be better rested and nourished, and sleep better in their own bed [4, 16, 37, 42]. In contrast, environmental comfort was lacking for hospital inpatients, and they complained about: the strange, busier and noisier ambience with a lot of activity going on in hospital; being confined to a certain amount of space; and lack of privacy and sleep disruption due to disturbances from other patients, nurses obtaining regular observations and new admissions etc. [4, 16, 52]. Generally, being in one’s own home was thought (by some of the UK healthcare professionals who were interviewed) to have promoted healing in a more holistic way with *“all the things that are important to him*” *(M3S2)*, i.e. patients were more satisfied with their sleep, diet, physical activity, stress level, social support, and environmental comfort, which was not possible in the hospital environment [4, 21].

*“It’s just a win-win to try and keep them at home and treat them at home. And less disorientating for them so, you know, especially elderly, frail or palliative patients, you know, we try and help them as much as we can and lessen that trauma of coming into hospital because it is quite a sort of busy, loud, noisy place. So all the advantages at home of having you know, your own cooked meal, and being with your pet dog and having your neighbour pop in, and your daughter, you know, it just really does show, you know, the benefits of being treated at home… the benefits far outweigh, you know, not getting sort of deconditioned in hospital and taken to their bed and you know, they’re not walking, they’re getting deep vein thrombosis, they’re getting pulmonary embolisms chest infections, you know, is definitely the way forward.” (M2S2)*

However, for some patients and carers, a key consideration was the potential for disruption to the rhythm and routines of patient’s home life [17, 39, 42]. This was minimised when visits were arranged at the agreed times that suited them or when staff clearly communicated the anticipated visiting times so that they could plan other activities such as meals, going out and having visitors, and when staff were reliable in following arrangements through [17, 39]. Conversely, high variability in care schedule and high staff turnover were regarded as real constraints [42]. Some carers reported experiencing no quiet time especially as there never was a fixed schedule and it was completely random, or that there were different people visiting them which they felt disturbing. For some, home storage of medical devices and materials was a problem when there was limited space in the home. For example, one carer reported that they “walked on each other’s feet” with all the materials, the wheelchair, the commode chair, the patient lift and the medical bed, leaving no space. It is apparent that these kinds of disruptions can disturb the equilibrium of the home environment and dampen its healing effects.

#### Better maintained physical and functional wellbeing

All the interviewed UK healthcare professionals as well as many patients and carers in the literature reported that patients returned to “normal”, i.e. baseline mobility and function, quicker than in hospital; and described how HaH enabled patients to maintain their mobility, activities of daily living and continuity in their established routines, which supported the maintenance of their independence [4, 16, 34, 52].

*“One of the key things is any other patient would be stuck in a hospital bed, that hospital bed would probably make them more stiff, more…more frail, more unwell. And quite often they’ll end up needing rehabilitation and all these other bits and pieces. But my gran’s up and about walking now [his grandma received HaH care].” (M7S1)*

In contrast, those in hospital were more limited in mobility and what activity they could do and described that the activities they were able to do were confined to stationary pursuits. In one study, patients requiring oxygen noted that the equipment provided within HaH allowed free movement while provision in hospital limited mobility [16]. In a service evaluation, carers reported that staff enabled patients to live as independently as possible by prescribing therapies and providing equipment that were suitable for patients in their home environment [34]. The evaluation also found that prompt diagnosis and delivery of appropriate medical interventions also contributed to improvement in patients’ functional ability.

#### Better maintained psychological and social wellbeing

Most patients reported that they were in a better mood, felt happier or less stressed, being in their own home [4, 16, 34, 42]. One study found that for patients with COPD, breathlessness was less marked despite higher activity levels at home, which could be associated with lower levels of anxiety as patients were more relaxed in the home and more content with HaH [16]. One carer looking after a patient with delirium appreciated avoiding additional distress in her mother that could have resulted from the unfamiliar surroundings of hospital and found it much easier to manage her confusion at home by using familiar cues to aide her memory [52]. Levine and colleagues found that many patients felt a general locus and sense of control surrounding one’s sleep, activity, nutrition, stress, and environmental comfort, and as interacting with professionals in the home resulted in care better tailored to one’s lived experience [4]. However, most patients were aware of the difficulties faced by their carers and felt guilty considering themselves a “burden” or a “weight”, which was a psychological burden to them [42].

HaH care was found to reduce the disruption to a person’s existing formal and informal care and support arrangements through the addition of acute-level care in their home; patients therefore were better able to maintain their usual social roles and activities and get social support, having family, friends and other support networks close by [1, 4, 16, 37]. This was also highlighted by some of the UK healthcare professionals who were interviewed. Home was found to be a more convenient place to meet family and friends; it was time and money saving and logistically easier for them, as travel, car parking, work absences, childcare issues and restrictive visiting hours etc. were avoided [4, 16].

*“I just remember a lovely wife that said to me, you know, we went out to do her husband’s intravenous antibiotics, because he had a very resistant bug. And you know, she said: ‘we’ve been married for, you know, 63 years, you know, I...I don’t want you to take him away from me. You know, his place is in this house. And you’ve come in and given him that treatment to get him better for me. And actually, that’s lovely, because now he still gets to see his grandchildren, the dog is still at home, you know, all the things that are important to him are still there, and he's...but he’s getting the treatment that he needs’.” (M3S2)*

#### Quicker and better recovery from acute problems

Some of the interviewed UK healthcare professionals as well as some carers in the literature noticed that patients’ acute symptoms (e.g. breathlessness and chest infection) improved considerably and sooner, and they perceived that patients’ recovery occurred more quickly during HaH [16, 17, 21, 34, 39].

*“We were all very pleasantly surprised by how well our patients did. We were treating fairly sick patients and they were getting...you know, they were improving probably more quickly than we would see in a hospital.” (M10S1)*

#### Patient’s and carer’s knowledge, skills, confidence and compliance strengthened in disease management and self-care

Patients and carers generally valued training, education and information support provided during HaH care [16, 34, 39, 42, 47]. Patient education that was empowering was perceived to be highly personalised and correspondent to the actual clinical situation and circumstances seen during the HaH care episode, e.g., specific advice related to medication, wound care or care plan [47]. It was also perceived to be comprehensive and understandable and have met patients’ and carers’ knowledge expectations at a “pace” acceptable to them. As a result, it increased patients’ ability and confidence in symptom management including treatment compliance and self-care, increased family carers’ knowledge and skills as care assistants, and increased both patients’ and family carers’ sense of control and safety, contributing to avoidance of possible clinical complications and hospital (re)admissions. These were also highlighted by some of the UK healthcare professionals who were interviewed.

*“We educate the patient so that they can continue that throughout the day…They’re managing their, and they’re able to manage their oxygen saturations and read some numbers off. And they actually then develop a certain sense of control and autonomy in their illness rather than being a kind of very passive participant in their illness in that…in the hospital bed.” (M1S3)*

However, lack of consistency was reported in one study when different team members told patients and carers different things on different visits, showing that they had not agreed on what should happen among themselves before talking to patients and carers [39]. In another study, patients felt that education received was fragmented, that is, while they appreciated education in certain areas, such as information about HaH care or further information about and feedback on their clinical condition, response to their actual knowledge expectations did not occur [47]. Rossinot and colleagues found that the lack of precise and realistic information on the practical functioning of HaH (particularly its pros and cons compared to hospital admission) before the decision of admission was made, resulted in some carers not realising the extent of involvement required of them and ending up feeling lost, disappointed, or deluded [42].

#### Carers better supported to fulfil caregiving role with minimum added care burden

Despite higher treatment needs of HaH patients, most carers did not report increased carer burden as had been anticipated; instead, some carers reported that hospital admission was more disruptive to them because of the time and organisation it took to do hospital visits and that hospital visits could be both physically and financially burdensome to them [17, 33, 38]; for some, the strain of extra caring work at home was balanced by the benefit of having greater understanding of and involvement in decision making around care [1, 4, 16], as reported by some of the interviewed UK healthcare professionals.

*“I think lots of our patients and carers have definitely felt that they’ve been more involved than they might have been if they’d come into hospital. Definitely having them at home, I think they’ve just found more reassuring, because they can be more involved in all of that decision making and caring, which might otherwise have been taken away from them, so you a short half hour visit, you know, every other day or something.” (M3S2)*

However, in two studies, some carers felt psychologically and emotionally burdened (e.g. sadness, helplessness) because of witnessing patient’s pain and suffering and because they also had to face the patient’s mood changes; some felt their workload strongly increased; and all these could lead to a deterioration of their relationship with patients as well as their own health and wellbeing [4, 42]. Vaartio‐Rajalin and colleagues found that patients' near‐ones could have mixed feelings, e.g. simultaneously feeling thankful, content and a relief that care was organised in the home, while also feeling their private space intruded, burdened and tired of their caregiving role and a need for respite [20, 21]. Therefore, it must be acknowledged that greater responsibility is required of family carers with HaH care and some are likely to experience some form of burden, whether it is emotional, physical, financial or other burden, and thus needing support.

Patients valued being treated in the home also because the added care of family members which was not possible in hospital; many relatives/carers felt that HaH staff had supported and enabled them to look after patients to the best of their ability as a carer [1, 17, 34, 39]. For example, when carers had experienced a flexible approach from HaH staff in responding to their relative’s extended care needs, especially during the challenging time following discharge from hospital, this had supported their own ability to cope and manage the patient [39]. Similar views were expressed by some of the UK healthcare professionals who were interviewed.

*“The carers are so reassured by having somebody coming in to check them that they don’t feel that it is entirely their responsibility anymore that they’ve got reassurance that we’re coming every day or twice a day to do the obs or make sure that they’re okay. I mean there are some carers who in particular hospital discharges I think where they don’t feel ready, and they don’t feel that they are able to do it. And that is us listening to them and supporting them as well as supporting the patient.” (M9S1)*

According to some of the interviewed UK healthcare professionals, feeling reassured that patients were provided with high quality professional care and thus safe with HaH team, carers and families reported having peace of mind during HaH care, for some this enabled them to continue working or taking a break from caregiving role [1, 34]. Some carers also appreciated not having to be separated from loved ones and being able to maintain their own daily routines [1, 34]. However, some had concerns around longer-term support which HaH could not provide. Some carers reported that HaH team had signposted them to community resources so that they could get help with their non-clinical needs (incl. emotional, financial, physical and social needs); and this can help improve their health and wellbeing and maintain their ability to care for patients.

#### Reduced care-seeking burden due to fragmentation and complexity of care system and logistics

Some interviewed UK healthcare professionals highlighted and some patients in literature reported better experiences with navigating the health care system, that is, more efficient processes and simplified logistics and continuity of care associated with admission, transfer, discharge, and generally access to care [4, 34, 41, 46, 52].

*“So if we’re giving IV antibiotics at home, for example, or IV Furosemide, they’re so pleased not to have to keep going back. Even when they’re just going back to the local hospital, you know, these are often really old, frail people who feel rubbish because they’ve got kidney problems, or heart failure, or whatever. And to just be able to stay at home in their own bed or in their own armchair, and have the treatment delivered at home, they’re delighted.”* (M9S1)

In one study [4], HaH patients described their admission as a seamless process, as they were often quickly transferred home from the ED, compared to a long wait for a hospital bed when they were admitted. Also, when care was delivered at home, by default care teams had to revolve around patients, as opposed to patients revolving around clinical teams in the hospital. As such, hospital inpatients in this study reported experiencing long waiting times and many administrative processes, which was burdensome particularly when one was ill, while HaH patients appreciated that they did not have to bear with such inconvenience and burden. Moreover, some HaH patients reported that to their surprise, HaH clinicians were indeed more available- whether by video, telephone, or home visits. They described having direct access to their home hospital clinicians at all hours of the day, compared with a call button and an uncertain wait for assistance in hospital, and that care providers appeared at their home surprisingly quickly when they were in need. In another study about rural HaH [41], patients living in remote rural areas particularly valued how this new model of care could remove the care seeking obstacles they would normally have to overcome to use hospital services, e.g. travelling to/from hospital (sometimes in extreme weather and road conditions) and waiting around for admission, daily rounds and discharge etc., and the associated time loss, discomfort, strain and unease. They appreciated the convenience, comfort and ease of using HaH that they had not experienced previously with hospital services. It was also found that due to the inconvenience, a few patients had often delayed seeking hospital care and having the option to receive hospital- level care at home, however, made them feel motivated again to seek timely care in future.

Transitioning from acute back to a community care setting with the associated care plan changes was regarded as a challenging time for patients and carers; HaH ensuring continuity of care through helping them re-connect with community services was regarded critical and highly valued; it could help them regain confidence and feel secure when they were faced with uncertainties following withdrawal of the acute service [4, 34, 52]. In a service evaluation [34], carers were impressed and pleased with HaH team’s excellent communication and the well-coordinated, joined up care they received; they felt that this had supported them in transitioning from hospital to community and made patients feel more secure realising that they had not “just been put out of hospital and abandoned”. However, in another study [4], for both HaH patients and hospital inpatients, discharge planning and the days following discharge were in general negative experiences. HaH patients cited difficulty carrying out the proposed plan after discharge, e.g. trouble obtaining medication. Inpatients faced similar problems with the added issue of adjusting to a new environment and new health care routines. In other studies, a cause for concern for some patients and carers came from lack of clarity over which healthcare services would be involved or available for any further problems after discharge from HaH service [39, 41].

#### Improved relational continuity of care through staff competency, consistency and investment in time and effort to build therapeutic relationships

Patients and carers gave predominantly positive feedback on HaH teams and they particularly valued better (relational) continuity of care through closer relationship or more regular contact with the same group of nursing, medical and therapy staff compared with inpatient care [4, 16, 21, 37]. Some patients in the literature described their relationship with HaH staff as “personal”, “individual” or being “more meaningful connections” [4, 16]. This closer relationship was also appreciated by the interviewed UK healthcare professionals. Conversely, lack of continuity disrupted rapport-building when many and inconsistent professionals had come to the home or was confusing to patients as remembering all the names and job titles was difficult [34, 39].

Moreover, patients and carers appreciated that compared to hospital staff, HaH staff were less rushed and spent more quality time with them during visits: listening to, observing, talking with them and providing care, which helped to build trust and contributed to staff members’ better understanding of their life and circumstances and making “a true assessment” of their needs [16, 17, 34]. For some carers, a closer relationship with and trust in HaH staff enabled them to share difficult experiences, and HaH staff listening to them lifted their mood when things were not going well [42]. Also, continuity through staff members taking time to understand the particular challenges for both patient and carer through sequential visits was valued and perceived to enable professionals’ meaningful monitoring of changes over time [39]. These were also appreciated by some of the interviewed UK healthcare professionals, regarded as advantages in providing person-centred care and which led to their higher job satisfaction, compared to their previous inpatient work.

*I think you get closer to the patients when you’re in the home because as I say, you get to see them in their own home environment, and obviously you get to see what their living conditions are like, you know, and you spend more time with them, so you get to know them better as they tend to open up to you more about other issues that are problematic which I don’t think if you’re on a ward, that they would, you know, they’d be like, oh, they’re too busy, you know, I'm not going to, but if you’re there, you know, and you’re giving them a treatment for like an hour, you know, they just open, they open up to you. (M2S1)*

Many patients and carers also praised the specialist expertise and the competency of the staff, citing that they appeared to be “well trained”, “remarkably competent”, “skilled and knowledgeable” and showed a high standard of care; and they expressed their confidence in the team [4, 16, 17, 34]. The words “kind”, “nice”, “friendly”, “supportive” and “caring” came up repeatedly within patients’ and carers’ comments on HaH staff as well as how they had felt cared about [4, 17, 34, 42]. There was also a general perception that HaH staff had good interpersonal and communication skills, they were thorough and capable of adapting to both the patient and environment’s unique requirements [4, 34, 39].

#### Sense of safety

Some patients and carers who declined HaH or received inpatient care in randomised controlled trials expressed doubts or worries about patient’s safety with HaH care- without “a cocoon of a hospital environment” or the proximity to care they were guaranteed by staying in the hospital [4, 16, 39]. For example, concern about the stability of their condition led to feelings of anxiety about HaH care, particularly when thinking about lack of rapid access to clinicians overnight. However, among those who had experienced HaH, there was a general feeling of safety among patients and carers, which was linked to them feeling “in very safe hands” and well supported by HaH teams [4, 16, 17, 34]. Two studies [4, 16] found that patients felt safe and reassured during HaH care due to: continuous vitals monitoring, daily visits from the nurses, the 24-h telephone support line, trust and confidence in the HaH clinical team, the availability of emergency services if return to hospital was needed, and the evening phone call (9 pm) to those patients living alone. Most patients were also not unduly concerned about potential delays in being seen by a doctor/clinician in the event of deterioration as they had experienced rapid access to HaH clinicians when in need as if in hospital. In addition, HaH patients cited the following as reasons for feeling safe: they felt they could call the care team anytime because of a closer relationship with them; the care team were more available whether by video, telephone, or visits and they had direct access to the team at all hours of the day, compared with a call button and an uncertain wait for assistance in hospital [4]. These findings echo those from the interviews.

*“It’s about making sure they’re safe and they feel well supported and the feedback we got from those patients were they felt really supported and they had that daily phone call, you know. And we could get them in, they were classed as inpatients on our service. So all our patients are classed as inpatients even though they’re at home, so we very much make that known to the patient that you are classed as an inpatient you’re just at home, so we can get you in quickly if we need to.” (M2S2)*

Patients noted that social support, whether in the form of family support or an aide provided by HaH service, was important to ensuring their safety [4]. Mäkelä and colleagues found that despite some families’ ‘rota’ system to sustain 24-h support during HaH care, there was apparent precariousness for the family in containing risks to patients (e.g. with dementia) at home [52]. Patients and carers felt they would need clear self-care and symptom management education to feel safe, and mentioned insufficient education about such issues, such as possible illness or treatment related limitations as reasons for feeling unsafe [47].

## Discussion

To our knowledge, this is the first attempt to systematically articulate and test a comprehensive programme theory for HaH. This has resulted in an integrated, overarching theory which encompasses three dimensions of HaH- organisation, utilisation, and impact, that are interlinked. The impact theory links effective care delivery and utilisation to the intended benefits, showing multiple, interacting pathways of change. It is central to the programme theory [24]: a programme must be resourced and organised in ways that make it possible to delivery services that can actuate the change processes leading to the intended impact; and the service delivery system must interact effectively with the target population to make it possible for them to receive and benefit from the services. Being clear about the (intentional and unintentional) effects of the programme’s activities and processes on service users and the change processes involved therefore can help inform and improve resourcing, organisation, delivery and utilisation.

A growing body of evidence has confirmed the predominantly positive impact of HaH on patients and carers and at the system level, and generally greater satisfaction of care compared to hospital admissions [4, 16, 17, 34, 39, 42, 52]. However, it remained unclear how the positive effects were brought about through service delivery and utilisation, e.g. exactly what HaH activities, processes, human interactions made the programme effective, safe and satisfactory to patients and carers and how. Our study has unpacked not only the multifaceted impact (benefits) of HaH care on patients and carers and beyond but also the change processes from delivering the required (essential) activities and functions to achieving the impact. Also highlighted are the key differences between acute home care and inpatient care and what contributes to the improved patient outcomes, experience and satisfaction at home: comprehensiveness of assessment leading to individually tailored, situational appropriate (realistic) interventions; special healing effects of home environment; high quality interactions between professionals and patients and carers; less complexity and fragmentation of care.

Our findings are particularly useful and timely for the current UK policy on virtual wards which include HaH services. The NHS has set out the ambition to implement virtual wards fully and as rapidly as possible, given the significant pressure on acute beds; and has asked local systems to develop detailed plans to optimise the rollout of virtual wards to deliver care for patients who would otherwise have to be treated in hospital [23]. We have provided policymakers with convincing evidence on patient and carer benefits of HaH to justify investment into HaH services. More importantly, we have unpacked the “black box” to reveal how these benefits can be brought about, which will help inform how HaH services can organise resources and design processes of care to optimise patient satisfaction and outcomes.

The main strength of our study is that drawing on both published evidence and empirical data, the combination of Framework Analysis [28] and Purposive Text Analysis [30] enabled us to not only identify shared components, features, ways of working across services but also unearth the underlying, complex interconnections and causal sequences among them. As the result, the theory provides insights into not only the organisational, utilisation and impact aspects of HaH but also the change processes from organisation, delivery and utilisation to benefits and impact. The impact theory is central to the programme theory: if the assumptions embodied in this component about how desired changes are effected by the HaH activities are faulty, or if they are valid but not well operationalised, the intended social benefits will not be realised [24].

As in other reviews adopting a realist logic [25, 26], we used multiple search strategies that made deliberate use of purposive sampling to retrieve materials fit for purpose in identifying, testing out or refining the programme theories. Within the limits of time and funding (one-year, rapid-response research with multiple, sequentially and conceptually linked components), we have assembled sufficient evidence (29 sources of evidence included) to satisfy the theoretical need. Nonetheless, we acknowledge that another review team may have made different judgements at key stages in the review process, e.g. criteria used to identify relevant sources of evidence and how to apply them to screen the sources, and judgments about the sources’ likely conceptual or descriptive contribution to the theory development. Therefore, while we endeavoured to include a wide variety of sources to articulate a comprehensive theory, we may have missed other potentially relevant sources of information that can influence how we develop the theory.

Another limitation is that in testing the soundness of the literature-based impact theory, service users’ experiences and views should have been explored to investigate whether the outcomes/impact are appropriate for the programme circumstances and are important and realistically attainable to the service users [24]. This was mainly due to the time and funding constraints of the study. Future researchers should conduct observations and interviews focusing on the target-programme interactions that are expected to produce the intended outcomes and crucially, service users’ perspectives should be included.

## Conclusions

We have identified multifaceted impacts of HaH on service users and the care system which add value to patient care, carer support and health system performance, thereby providing convincing evidence that HaH is a better option for some patients (particularly older people) who would otherwise need hospital admission. Our findings also highlight the main features of HaH that contribute to patients’ better physical, functional, and psychosocial wellbeing and better general experience and satisfaction: more holistic and individually tailored professional care; more holistic healing effects of home environment; better interactions and therapeutic relationships between professionals and patients and carers; and less complexity and fragmentation of care.

We have made the first ever attempt to systematically articulate and test an overarching programme theory for HaH, which consists of the organisational theory, the utilisation theory and the impact theory. The impact theory helps inform how HaH services can organise resources and design processes of care to optimise patient satisfaction and outcomes. Further research should focus on barriers faced by HaH services in adopting the organisational configurations and care processes highlighted in this study. Patients and their carers should perceive the impact of the organisational changes and therefore their experiences will determine how successfully these changes have been implemented. The collection of know-how we have created can be used as a basis for formulating and prioritising evaluation questions, designing evaluation research, and interpreting evaluation findings in future HaH service evaluations.

### Supplementary Information

**Additional file 1. **Research process and rationale.

**Additional file 2. **Criteria for identifying sources and their application_HC31Oct2023.

**Additional file 3. **Details of included papers.

**Additional file 4. **4 NVivo Codebook for theory development.

**Additional file 5. **Interview topic guide.

**Additional file 6. **Consent text in email.

**Additional file 7. **Background information about participants.

## Data Availability

The datasets generated and analysed during the current study are available from the corresponding author on reasonable request.

## Abbreviations

HaH: Hospital at Home
NHS: The National Health Service
MDT: Multidisciplinary Team
