# Supplementary material for: An integrated understanding of the impact of hospital at home: a mixed-methods study to articulate and test a programme theory
Source: BMC Health Serv Res. 2024 Feb 2;24:163. doi: 10.1186/s12913-024-10619-7 (PMC10835828; doi:10.1186/s12913-024-10619-7)
Supplement: Supplementary file 1 — Additional file 1. Research process and rationale. [file 12913_2024_10619_MOESM1_ESM.docx]

**Main outputs**

**Outcomes**

**Components**

- 29 articles included
- Draft programme theory and review findings
- Improved topic guide for workshops (B)
- Early drafts of survey (C)

**A.** Review & synthesis of existing evidence

***Objective 1***

HaH programme theory

- Six surveys completed by six services included into analysis
- Fixed costs (annual, monthly and per patient) associated with staffing calculated and compared between services
- Framework for developing a national data registry based on theory and survey results
- Survey finalized and hosted on Qualtrics
- Survey completed by 49 respondents
- Survey data analysed using descriptive statistics
- Survey data used for economic analysis (D)
- 13 interviews with 16 participants from 11 services
- Draft theory from review (A) tested using workshop findings
- Implementation/adaptation lessons learnt
- Resources and costs data collected for economic assessment (D)
- Survey draft (C) improved using workshop findings

**B.** HaH professional interviews

**C.** Web-based provider baseline survey

***Objective 3*** Current situation of UK HaH provision

***Objective 4***

Resource use & associated costs/savings

***Objective 5***

National data registry design & IT set-up

***Objective 2*** Service adaptation/set-up lessons

**E.** Evidence synthesis and application

**D.** Assessment of comparative resource costs
