# Supplementary material for: An integrated understanding of the impact of hospital at home: a mixed-methods study to articulate and test a programme theory
Source: BMC Health Serv Res. 2024 Feb 2;24:163. doi: 10.1186/s12913-024-10619-7 (PMC10835828; doi:10.1186/s12913-024-10619-7)
Supplement: Supplementary file 2 — Additional file 2. Criteria for identifying sources and their application_HC31Oct2023. [file 12913_2024_10619_MOESM2_ESM.docx]

*Types of home-based services to be excluded:*

| - Enhanced primary care provision (e.g. GP extensivist roles or Virtual Wards); - Ongoing chronic disease management by primary care team and outpatient specialist team; - Intermediate care for the management of medically stable patients; - Day Care for the management of stable ambulant patients; - Admission prevention programmes focusing on chronic disease management and high-risk categories such as frailty to prevent decline leading to admission. - Palliative/end-of-life care provision by specialist palliative/end-of-life services. |
| --- |

*Working definition of Hospital at Home^[[1]](#footnote-1)^ used to differentiate Hospital at Home services from other home-based services:*

| **Purpose** | To provide alternatives to traditional admission for patients who are acutely unwell and would normally require an acute hospital bed. |
| --- | --- |
| **Functions** | - Management of patients with level of acuity and complexity that cannot be managed safely and effectively by other community services. - Time limited short-term intervention of 1-14 days. - Patient and family centred care in partnership with the team. - An adjunct to, and compliment, other community-based healthcare initiatives which support patients to remain in their own home. |
| **Structure** | - The individual to be admitted to an acute care team and under clinical governance as though in hospital. - Secondary care level specialist leadership and clear lines of clinical responsibility. - Defined inclusion and exclusion criteria, with defined target population for example over 18 or over 65. - Patients to have equity of access to other specialty advice as though an inpatient. |
| **Content** | - Access to hospital-based lab and imaging services as if patients were in hospital and may also use portable diagnostic machines/devices that can be carried into the patient’s house to run point-of-care (POC) diagnostics. - Hospital level interventions (such as access to intravenous fluids, therapy and oxygen). - Psychological, practical and social support. |
| **Delivery** | - Patients treated as though admitted to hospital but managed within their own home. - Daily input from a multidisciplinary team and sometimes multiple visits and provisions for 24-hour cover with the ability to respond to urgent visits. |

*Application of the criteria:*

At the outset of screening the titles and abstracts of the sources identified through database searches, the aim was to identify all sources of evidence potentially related to HaH and exclude all sources related to other type of home-based services. Sources were included if they described, evaluated, or studied a model of HaH or a HaH service. A broad definition of HaH was used, i.e. the service must deliver care in private or care *homes* to treat *acutely* unwell, *adult* patients who would normally require an *acute* hospital bed. There were times when the above criteria were used by the reviewer to facilitate the judgement; if still unclear, other members of the research team were consulted to reach a group decision or occasionally full-text sources were screened.

At the full-text assessment stage, rather than strictly applying the above working definition of HaH, the reviewer formed a judgement *as a whole* on whether or not a source was includable allowing for varieties and adaptations of HaH in different contexts, even if a source did not exhibit all the characteristics described above. When the reviewer was unable to make a decision to include or exclude, other members of the research team were consulted to make a group decision.

The literature review was time and conceptually critical for developing subsequent components, particularly the professional interviews and the provider survey (reported elsewhere). Therefore, we used a single reviewer (different at different stage) for screening records, selecting papers, and extracting data, without verification by a second reviewer. Also, some of the tasks involved were done by the two single reviewers (first and second authors) in parallel, i.e. while the first author selected papers from those accumulated prior to the study and then started to extract data from these papers, the second author conducted searches and screened new records using predefined criteria.


1. <https://www.hospitalathome.org.uk/what-is-hospital-at-home> [↑](#footnote-ref-1)
