# Supplementary material for: An integrated understanding of the impact of hospital at home: a mixed-methods study to articulate and test a programme theory
Source: BMC Health Serv Res. 2024 Feb 2;24:163. doi: 10.1186/s12913-024-10619-7 (PMC10835828; doi:10.1186/s12913-024-10619-7)
Supplement: Supplementary file 3 — Additional file 3. Details of included papers. [file 12913_2024_10619_MOESM3_ESM.docx]

| **Author** | **Publication Year** | **Country** | **Type of document** | **Research method** | **Title** |
| --- | --- | --- | --- | --- | --- |
| Farfan-Portet M-I, Denis A, Mergaert L, Daue F, Mistiaen P, Gerkens S. | 2015 | Belgium | Scientific Report | Literature review + interviews + stakeholder workshop | Hospital at Home: orientations for Belgium – Short report. |
| Healthcare Improvement Scotland | 2020 | Scotland | National service guideline | Literature review | Hospital at Home  Guiding principles for service development January 2020 |
| Ilaria Amantea, Marzia Arnone, Antonio Di Leva, Emilio Sulis, Dario Bianca, Enrico Brunetti, and Renata Marinello | 2019 | Italy | Research paper | Business Process Modelling and Notation | Modeling and Simulation of the Hospital-at-Home Service Admission Process. |
| Stephen Bourke John Steer  Carlos Echevarria  Elizabeth Norman | 2017 | England | Service Manual  (Supplementary file of a research paper) | NA | Hospital at Home Manual |
| Brody, A.A., Arbaje, A.I., DeCherrie, L.V., Federman, A.D., Leff, B. and Siu, A.L. | 2019 | US | Research paper | Focus group + interviews | Starting Up a Hospital at Home Program: Facilitators and Barriers to Implementation. |
| Bryant, P.A., Rogers, B.A., Cowan, R., Bowen, A.C., Pollard, J. and Hospital‐in‐the‐Home Society Australasia | 2020 | Australia | Position paper | NA | Planning and clinical role of acute medical home care services for COVID‐19: consensus position statement by the Hospital‐in‐the‐Home Society Australasia. |
| Burns, V., Misra, V. and Paton, N. | 2020 | UK | Evaluation paper | NA | Systemic anti-cancer therapy delivery in the home: a service model. |
| James Burton, Matthew Graham-Brown | 2017 | UK | Evaluation Report | NA | Designing and implementing an acute hospital at home service. |
| DeCherrie, L.V., Wajnberg, A., Soones, T., Escobar, C., Catalan, E., Lubetsky, S., Leff, B., Federman, A. and Siu, A., 2019. | 2019 | US | Research paper | Service design + medical record review | Hospital at home‐plus: a platform of facility‐based care. |
| Dismore, L.L., Echevarria, C., Van Wersch, A., Gibson, J. and Bourke, S., 2019. | 2019 | UK | Research paper | Interview | What are the positive drivers and potential barriers to implementation of hospital at home selected by low-risk DECAF score in the UK: a qualitative study embedded within a randomised controlled trial. |
| Facultad, J. and Lee, G.A. | 2019 | UK | Research paper | Questionnaire | Patient satisfaction with a hospital-in-the-home service. |
| Gardner, M., Shepperd, S., Godfrey, M., Mäkelä, P., Tsiachristas, A., Singh-Mehta, A., Ellis, G., Khanna, P., Langhorne, P., Makin, S. and Stott, D.J. | 2019 | UK | Research report | Cochrane review + survey + medical record review + interviews + case study + Delphi method | Comprehensive geriatric assessment in hospital and hospital-at-home settings: a mixed-methods study. |
| Gray, E., Currey, J. and Considine, J. | 2018 | Australia | Research paper | Literature review | Hospital in the home nurses’ assessment decision making: An integrative review of the literature. |
| Hernández, C., Aibar, J., Seijas, N., Puig, I., Alonso, A., Garcia-Aymerich, J. and Roca, J. | 2018 | Spain | Research paper | Service design + medical record review | Implementation of home hospitalization and early discharge as an integrated care service: a ten years pragmatic assessment. |
| Jester, R., Titchener, K., Doyle-Blunden, J. and Caldwell, C. | 2015 | UK | Research paper | Literature review | The development of an evaluation framework for a Hospital at Home service: Lessons from the literature. |
| Karacaoglu, K. and Leask, C. | 2019 | UK | Evaluation report | Medical record review + survey + interviews | Acute Care at Home (AC@ H) Test of Change. |
| Levine, D.M., Pian, J., Mahendrakumar, K., Patel, A., Saenz, A. and Schnipper, J.L. | 2021 | US | Research paper | Interviews | Hospital-level care at home for acutely ill adults: a qualitative evaluation of a randomized controlled trial. |
| Levine, D.M., Paz, M., Burke, K. and Schnipper, J.L. | 2022 | US | Research paper | Medical record review | Predictors and reasons why patients decline to participate in home hospital: a mixed methods analysis of a randomized controlled trial. |
| Levine, D.M., Desai, M.P., Ross, J.B., Como, N. and Holley, S. | 2021 | US | Research paper | Service design/simulation + observation + interviews | Scoping and testing rural acute care at home: a simulation analysis. |
| Levine, D.M., Desai, M.P., Ross, J., Como, N. and Anne Gill, E. | 2021 | US | Research paper | Interviews + focus group | Rural perceptions of acute care at home: a qualitative analysis. |
| Mäkelä, P., Stott, D., Godfrey, M., Ellis, G., Schiff, R. and Shepperd, S. | 2020 | UK | Research paper | Interviews | The work of older people and their informal caregivers in managing an acute health event in a hospital at home or hospital inpatient setting. |
| Morano, B., Jimenez-Mejia, J., Sanon, M., Morano, C., DeCherrie, L.V. | 2020 | US | Book chapter | NA | Acute Care in the Home Setting: Hospital at Home. |
| Guys & St Thomas NHS Foundation Trust (Community Health Services) | 2015 |  | Service guidance | Report | Integrated Care Clinical Pharmacist for Frail Older People: Case Management and Enhanced Rapid Response. |
| Department of Health of Queensland Government | 2017 | Australia | Service guidelines | NA | Hospital in the Home Guidelines |
| Rossinot, H., Marquestaut, O. and de Stampa, M. | 2019 | France | Research paper | Interviews | The experience of patients and family caregivers during hospital-at-home in France. |
| Siu, A.L., Zimbroff, R.M., Federman, A.D., DeCherrie, L.V., Garrido, M., Morano, B., Lubetsky, S., Catalan, E. and Leff, B. | 2019 | US | Research paper | Questionnaire | The effect of adapting Hospital at Home to facilitate implementation and sustainment on program drift or voltage drop. |
| Vaartio‐Rajalin, H., Ngoni, K. and Fagerström, L. | 2020 | Finland | Research paper | Focus groups | Balancing between extremes—Work in hospital‐at‐home. |
| Vaartio‐Rajalin, H. and Fagerström, L. | 2019 | Finland | Research paper | Literature review | Professional care at home: patient‐centredness, interprofessionality and effectivity? A scoping review. |
| Vaartio-Rajalin, H., Nyholm, L. and Fagerström, L. | 2020 | Finland | Research paper | Interviews | Patient education in the hospital-at-home care context. |
