# Supplementary material for: An integrated understanding of the impact of hospital at home: a mixed-methods study to articulate and test a programme theory
Source: BMC Health Serv Res. 2024 Feb 2;24:163. doi: 10.1186/s12913-024-10619-7 (PMC10835828; doi:10.1186/s12913-024-10619-7)
Supplement: Supplementary file 5 — Additional file 5. Interview topic guide. [file 12913_2024_10619_MOESM5_ESM.docx]

**How to utilise the potential of Hospital at Home to deliver more acute non-COVID and COVID care outside of hospital**

**Topic Guide** (Stage 1)

| 1. What are the main features of your service, particularly those distinguishing your service from other home-based services?  - *Target population / characteristics of patient groups, e.g. medical conditions, health and functional status, age group, gender, ethnicity, socioeconomic status, carer situation, home environment* - *Type of HaH, e.g. admission avoidance, early discharge* - *Clinical responsibility, e.g. GP led, specialist led* - *Funding source(s)* - *Multidisciplinary care, e.g. all members of MDT provided internally or in partnership with external services*  1. How and from where are eligible patients referred to your service?    - *Patients without COVID and patients with COVID*    - *Criteria and support to referrers*    - *Missed referrals e.g. out of hours and weekends, and solutions* 2. What are the processes involved in delivering usual (non-COVID) service from admission to discharge?  - *Process of admission, e.g. assessment, factors to consider in admission decision-making* - *Operation hours/Out-of-hours arrangement for patients* - *Diagnostics, e.g. hospital-based lab and imaging, point-of-care* - *Monitoring (what is monitored and how)* - *Medical and non-medical reviews* - *Acute medical treatment and nursing care – pharmacy support and drug delivery* - *Escalation and emergency response* - *Allied Health and supportive care, e.g. therapists’ and social care input* - *Patient and carer education and information support* - *Discharge process* - *MDTs, functions of electronic health records support delivery or not* - *Adaptations/tailoring to individual needs and home life, e.g. location, space, routines, relationships (incl. therapeutic relationships)* - *Differences between patients at home and in care homes*  1. What are the clinical pathways and processes of care for COVID patients?  - *Creating staff capacity* - *Reducing staff and patients’ exposure to COVID* - *Access to COVID-related treatment, e.g. oxygen, drugs, palliative care* - *Involvement of family/carers* - *Differences between patients at home and in care homes*  1. What are the expected outcomes of your current model of care? 2. How do you monitor progress against your expected outcomes? What data do you routinely collect on patients?  - *What data or information, if any, would you have liked to have collected, but couldn’t? Why?*  1. What factors hindered or supported the implementation/adaptation of your Hospital at Home service during the pandemic?  - *Selection of patient groups for service* - *Establishing/maintaining referral pathways, e.g. GP/ambulance engagement* - *Establishing/coordinating partner services to provide multidisciplinary care, e.g. capacity, quality and timeliness of external services* - *Documenting and sharing activity/patient information across interfaces* - *Staffing model/capacity, e.g. unpredictable and immediate enrolment needs of patients versus stafﬁng model* - *Pharmacist’s input* - *Patient/carer involvement* - *Reimbursement system and its limitations*  1. What are the lessons learnt from implementing/adapting the service during the pandemic?    - *Benefits and limitations of the model*    - *Scalability*  - *Addressing inequalities in patient access, e.g. influence of psychosocial, housing, economic and cultural contexts* - *Addressing safety concerns of professionals and service users and creating realistic expectations* |
| --- |
