# Supplementary material for: An integrated understanding of the impact of hospital at home: a mixed-methods study to articulate and test a programme theory
Source: BMC Health Serv Res. 2024 Feb 2;24:163. doi: 10.1186/s12913-024-10619-7 (PMC10835828; doi:10.1186/s12913-024-10619-7)
Supplement: Supplementary file 6 — Additional file 6. Consent text in email. [file 12913_2024_10619_MOESM6_ESM.docx]

RE: Participant Information Sheet, Consent and your Availability

Dear ……

Thank you very much for expressing your interest in participating!

*Please note that workshop 1 (or interview 1) will be held from 13^th^ to 26^th^ September 2021 and Workshop 2 (or interview 2) from 11^th^ to 24^th^ October 2021. You will have more flexibility in choosing when to participate including in the evenings and over weekends.*

This email includes:

- A consent form in the body text below.
- Participant Information Sheet (as an attachment).
- A form asking simple questions about you and your availability for the workshops (as an attachment).

If you are happy to take part after reading the attached Participant Information Sheet, **please** **reply directly to this email: with the consent form below completed and the form (about you and your availability) completed and attached.***Please reply within 5 days of receiving this email!*

We will then contact you to let you know the date and time of the workshops and the information you will need to join the workshops.

| **CONSENT FORM**  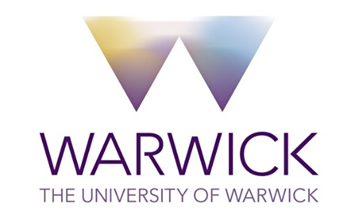**Title of Project:**   How to utilise the potential of Hospital at Home to deliver more acute non-COVID and COVID care outside of hospital  **Name of Researcher(s):** Professor Daniel Lasserson, Dr Hong Chen, Dr Terry Brown, Professor Richard Lilford (University of Birmingham), Dr Agnieszka Ignatowicz (University of Birmingham), Dr Magdalena Skrybant (University of Birmingham)  ***To give your consent, please 1) type 'YES’ after each statement*** *(see example below)****, and 2) type your name and date at the end of the form!***   1. I confirm that I have read and understand the information sheet [Version2.0, 22/06/21] for the above study. I have had the opportunity to consider the information, ask questions and have had these answered satisfactorily. YES 2. I understand that my participation is voluntary and that I can withdraw from the study at any time without giving an explanation and with no disbenefit. I also understand that due to the nature of the group discussion, during and after the workshops, my data cannot be withdrawn. 3. I understand that data collected during the study, may be looked at by individuals from The University of Warwick and The University of Birmingham, or from regulatory authorities, where it is relevant to my taking part in this study.  I give permission for these individuals to have access to my data. 4. I understand that part of this research involves recording data. These will be kept securely and stored separately to any identifiable information, i.e. consent forms and contact details. I agree to being recorded. 5. I agree to take part in the above study.   **Name of Participant:**  **Date:** |
| --- |

Many thanks! And please let us know if you have any questions.

Hong

**Dr Hong CHEN** BMS  MPH  MSc  PhD

Research Fellow

NIHR ARC West Midlands

Division of Health Sciences

Warwick Medical School

University of Warwick

[*hah@warwick.ac.uk*](mailto:hah@warwick.ac.uk)
