# Supplementary material for: An integrated understanding of the impact of hospital at home: a mixed-methods study to articulate and test a programme theory
Source: BMC Health Serv Res. 2024 Feb 2;24:163. doi: 10.1186/s12913-024-10619-7 (PMC10835828; doi:10.1186/s12913-024-10619-7)
Supplement: Supplementary file 7 — Additional file 7. Background information about participants. [file 12913_2024_10619_MOESM7_ESM.docx]

| Total number of interviewees: **16** | | | | |
| --- | --- | --- | --- | --- |
| **Interviewee code:**  **M (service model)**  **S (speaker)** | **Profession** | **Clinical training background** | **Years worked at Hospital at Home** | **Role and main responsibilities in Hospital at Home** |
| M2S2 | Nurse | BSC (Hons) in Adult Nursing graduated in 2001.  Advanced History taking level 7. Diagnostic Reasoning level 7. Non-medical prescriber V300. | 4.5 | Senior Specialist Nurse Practitioner responsible for delivering care to a group of patients as per referral at band 7 level including admission avoidance. Helping develop the Hospital at Home service and supporting team development. |
| M5S1 | Physiotherapist | MSc Clinical diagnostics and Examination. | 1 | Team Leader for Hospital at Home. |
| M6S1 | Occupational therapist | Ms in OT Advancing Practice. | 5 | Managing own caseload as loan worker. Specialized in restoring elderly loss of function/preventing further functional decline by providing short term rehabilitation/equipment/coordinating care and referring to community services if required. Supervising and appraising junior staff. |
| M6S2 | Doctor | Specialty Doctor (Staff Grade).Completed foundation training and core medical training. Worked as a registrar in Endocrine and Diabetes for 2 years before leaving the training post. | 3 | Triaging referrals, assessing patients on admission and commencing treatment. Arranging additional investigations as needed. Contributing to twice daily consultant led MDT meetings about patients. |
| M2S1 | Nurse | Advanced Nurse Practitioner. District Nurse | 5 | Operation Lead. Lead to ANPs for Hospital at Home. |
| M3S1 | Nurse | Nursing Degree and Registered SNP in Cancer and Palliative Care. 21 years’ experience in Nursing including MAU/EAU, Acute Medicine, Gerontology, Oncology, Palliative care, Ambulatory/AAU, CC@H, COPAT and Hospital at Home. QSIR and GCP qualified. | 5 | Lead Nurse for Hospital at Home X, Hospital at Home Y, Covid Care @ Home and COPAT. Responsible for running of the services and for service design and innovation. |
| M7S1 | Doctor | Acute physician. | 1 | Developing Hospital at Home model for patients across X county. |
| M10S1 | Doctor | Consultant geriatrician. | 7 | Clinical Lead for Acute Care at Home and direct patient care. |
| M8S1 | Doctor | Medical training post but then CCTR in General Practice. Now working full time in Geriatrics. | 3.5 | Co-Lead for Hospital at Home service, Senior clinician for service Mon-Thursday  Development of service: responsibilities in patient engagement, ACP, CCF, MDT working. |
| M1S1 | Physician Associate |  | 1.5 | Running of AMU Virtual Ward at X hospital and admin work. |
| M1S2 | Doctor | Middle grade doctor. |  | Running of AMU Virtual Ward and in charge of PAs. |
| M1S3 | Doctor | Middle grade doctor. |  | Running of AMU Virtual Ward and in charge of PAs. |
| M4S1 | Nurse | Community Nursing. | 2 | Clinical Development Lead - main role, clinically developing the ward, responsible for ensuring the advanced practitioners (Community Matrons) and other community staff have access to the training they need, development of required clinical pathways, reviewing patient cases, data and incidents, liaising with referrers to encourage referrals to avoid unnecessary hospital admissions etc. |
| M3S2 | Doctor | Consultant Geriatrician with CCT in Geriatrics & general medicine. | 1.5 | Consultant and clinical lead for Hospital at Home service. Director for Hospital at Home service in X region. |
| M11S1 | Doctor | MBChB, PhD, FRCP. | 1.5 | Lead consultant |
| M9S1 | Nurse | Registered nurse, Specialist Practitioner -District nursing and newly qualified Independent Prescriber. | 10 | Clinical lead for Hospital at Home South team in X county and RN carrying out visits for assessment and treatment. |
